# The Immune Response to SARS-CoV-2 Vaccination: Insights Learned From Adult Patients With Common Variable Immune Deficiency

**DOI:** 10.3389/fimmu.2021.815404

**Published:** 2022-01-19

**Authors:** Isabella Quinti, Franco Locatelli, Rita Carsetti

**Affiliations:** ^1^ Department of Molecular Medicine, Sapienza University of Rome, Rome, Italy; ^2^ Department Onco-Haematology, and Cell and Gene Therapy, Bambino Gesù Children Hospital, Istituto di Ricovero e Cura a Carattere Scientifico (IRCCS), Rome, Italy; ^3^ Dipartimento Materno-Infantile e Scienze Urologiche, Sapienza University of Rome, Rome, Italy; ^4^ Diagnostic Immunology Research Unit, Multimodal Medicine Research Area, Bambino Gesù Children’s Hospital, Istituto di Ricovero e Cura a Carattere Scientifico (IRCCS), Rome, Italy; ^5^ Diagnostic Immunology Clinical Unit, Bambino Gesù Children’s Hospital, Istituto di Ricovero e Cura a Carattere Scientifico (IRCCS), Rome, Italy

**Keywords:** immunization, common variable immune deficiency, vaccine, antibodies, SARS-CoV-2

## Abstract

CVID patients have an increased susceptibility to vaccine-preventable infections. The question on the potential benefits of immunization of CVID patients against SARS-CoV-2 offered the possibility to analyze the defective mechanisms of immune responses to a novel antigen. In CVID, as in immunocompetent subjects, the role of B and T cells is different between infected and vaccinated individuals. Upon vaccination, variable anti-Spike IgG responses have been found in different CVID cohorts. Immunization with two doses of mRNA vaccine did not generate Spike-specific classical memory B cells (MBCs) but atypical memory B cells (ATM) with low binding capacity to Spike protein. Spike-specific T-cells responses were also induced in CVID patients with a variable frequency, differently from specific T cells produced after multiple exposures to viral antigens following influenza virus immunization and infection. The immune response elicited by SARS-CoV-2 infection was enhanced by subsequent immunization underlying the need to immunize convalescent COVID-19 CVID patients after recovery. In particular, immunization after SARS-Cov-2 infection generated Spike-specific classical memory B cells (MBCs) with low binding capacity to Spike protein and Spike-specific antibodies in a high percentage of CVID patients. The search for a strategy to elicit an adequate immune response post-vaccination in CVID patients is necessary. Since reinfection with SARS-CoV-2 has been documented, at present SARS-CoV-2 positive CVID patients might benefit from new preventing strategy based on administration of anti-SARS-CoV-2 monoclonal antibodies.

## Introduction

The Committee of Experts on Primary Immunodeficiency of the International Union of Immunological Societies (IUIS) has included vaccination both as a diagnostic tool to assess the specific antibody response to protein and/or polysaccharide antigens and as a means of prevention (1).

The type and severity of the immunodeficiency determines the efficacy of vaccines, with varying levels of impairment, ranging from normal as in immunocompetent individuals, to incomplete or even absent. The degree of immunodeficiency and the specific defect in antibody production are variable in common variable immunodeficiency (CVID) (2) and each patient should be studied as unique in terms of cellular and humoral responses.

The decision to vaccinate a patient must include a risk and benefit assessment to ensure maximum protection and avoid adverse events. In addition, other factors, including the type of vaccine, the interval between administrations, and the time between gamma globulin administration and vaccination, must also be taken into account in defining an immunization strategy.

Here, we provide an updated perspective on the pathogenesis of CVID based on the studies performed on immune responses to vaccines with the aim to evaluate whether the immunization strategy for adult patients with CVID is effective. Studies on the potential benefits of immunization against SARS-CoV-2 offered the possibility to investigate the impaired pathogenic mechanisms of response to a novel antigen in patients with CVID.

## Susceptibility to Vaccine-Preventable Infections

CVID patients have an increased susceptibility to vaccine-preventable infections. Although the predominant infections are of bacterial origin, viral infections caused by rhinoviruses, parainfluenza, noroviruses, and herpesviruses, including varicella herpes zoster (VZV), adenovirus, respiratory syncytial virus, that, in turn, play a role in driving an underlying inflammatory condition, are reported in CVID (3). SARS-CoV-2 infection also has been reported in CVID, since the beginning of the pandemics, with a low prevalence possibly due to the choice of most physicians to inform CVID patients early about safety measures, and to switch most patients to home therapy and remote assistance (4). Within SARS-CoV-2 infected patients with inborn errors of immunity, CVID patients represent the largest proportion since CVID is the most commonly diagnosed/reported IEI (5), have more comorbidities and older age (5). Moreover, CVID patients have an increased risk for prolonged infections and a low probability to clear viruses as it has been demonstrated for SARS-CoV-2 (6) as well as for the poliovirus, in particular when the number of peripheral blood B lymphocytes is low (7). Genetic differences contribute to individual variations in the immune response to pathogens and in the response to immunization. Pathogenic loss-of-function or gain-of-function heterozygous variants have been reported to be associated with CVID. However, their functional relevance for susceptibility to infection and response to vaccination remains to be clarified (8). Unfortunately, genetic causes of most CVID cases remain undefined, and the diagnosis is predominantly based on hypogammaglobulinemia with impairment of antibody response to vaccine or natural antigen and reduced memory B cells (MBCs) frequency.

Few data are available on genetic factors associated with the impaired SARS-CoV-2 response in CVID. A CVID patient with NF-kB2 loss-of-function variant who developed severe COVID-19 and a patient with TBK1 and TNFRSF13B mutations and an auto-inflammatory disease with lethal COVID-19 were reported (9, 10).

**Table 1** illustrates immune alterations found in patients with CVID relevant for the impaired and variable response to vaccines (2). In the majority of patients, CVID is associated with defects in late stages of B cell development. In a group of CVID patients, plasma protein profiling identified a Th1-driven immune dysregulation, with increased plasma levels of IFN-γ and of proteins regulated by IFN-γ, NF-kB2 and NF-kB1 haploinsufficiency, or increased plasma levels of CXCL13 reflecting aberrant germinal center (GC) formation (12).

**Table 1:** Immune alterations relevant to impaired response to vaccines in CVID.

Immune function	Abnormality
Immunoglobulin levels	Reduced or absent
Bone marrow plasma cells	Depleted
Germinal Centre reaction	Impaired
Stimulation *via* Toll like receptors 7, 8, and 9 in B cells and/or plasmacytoid dendritic cells	Impaired
Switched memory B cells	Reduced or absent
Circulating CD4 T cells	Reduced
Naive CD4 T cells	Reduced
Proliferation and activation of antigen-specific T cells	Impaired
CD40L expression	Reduced
T follicular helper cells	Decreased
T-cell receptor repertoires	Restricted
IL-2, IL-10	Reduced
IL-6	Increased
Thymic maturation	Impaired
Monocyte/dendritic cell	Defective function
Innate immune responses	Impaired function
NK cells	Reduced

Bonilla et al., 2016 (11).

Aberrant germinal center reactions are also observed in previously healthy individuals affected by severe COVID-19. In these cases, the excessive reaction of the innate immune system with production of high concentrations of inflammatory cytokines disrupts the architecture of the germinal center thus preventing the response (13). The impaired germinal center reaction in COVID-19 leads to the generation of extrafollicular responses and increase of atypical memory B cells (14).

Thus, the inability to perform the germinal center reaction genetically determined in CVID, is induced by the explosive innate immune reaction to SARS-CoV-2 in severe COVD-19.

## Vaccinations in CVID

As stated above, immunization can be used in patients with impaired and residual B-cell function to provide information on specific humoral immunity and to improve the outcomes related to vaccine-preventable disease. In CVID patients, vaccination is a tool to evaluate T-dependent and T-independent antibody residual function of B lymphocytes and it might be used to measure specific cellular immunity (15). Thus, vaccination has both a therapeutic and diagnostic role, but might be also useful to predict the prognosis (16). In fact, the inability to mount a response against the pneumococcal polysaccharide antigens or the inability to maintain the antibody response over time identified CVID patients with a severe immunological impairment. These patients have a great risk of comorbidities and poor prognosis. Alternative or complementary measurements of other polysaccharide responses have also been proposed, in an attempt to increase the diagnostic accuracy. For example, responses to the less frequently used *Salmonella typhi* pure polysaccharide vaccine (Typhim Vi) have been studied. In a multicenter study, Guevara-Hoyer et al. (17) demonstrated a lack of response in both Typhim Vi and pneumococcal immunization in a group of CVID patients, suggesting that the evaluation of the specific antibody response to Typhim Vi vaccine may add clinical value to the diagnosis of impaired anti-polysaccharide antibody production in CVID.

More detailed information is available on immune responses to vaccines against viral infections, including influenza and VZV vaccination.

Influenza vaccines provide protection by generating high-affinity antibodies against viral hemagglutinin. High-affinity antibodies are produced during the germinal center (GC) reaction through the interaction between T follicular helper cells and B cells. The response to vaccination can be measured by the increase of hemagglutination-inhibiting antibodies and specific T-cell responses, demonstrated by the presence of vaccine-induced CD4 T cells and cytokine production. CVID patients with switched MBCs (Euroclass smB+) (18) were shown to have T-cell cytokine responses to vaccines comparable to those of healthy controls, but the vaccine specific antibody responses were found impaired in the group of patients with a more severe B-cell defect. As an alternative readout for the effective T-cell response to vaccination, it is possible to identify antigen-specific T cells by evaluating the upregulation of CD25 and CD134 (OX40) following *in vitro* re-stimulation with vaccine-derived peptides (19). VZV is the only human herpesvirus for which highly effective vaccines are available. The recent development of a liposome-based (HZ/su) subunit vaccine, which contains VZV, glycoprotein E and the adjuvant ASO1B, promises to change the perspectives for immunization against herpes zoster and its complications in adult CVID patients for whom a live-attenuated vaccine is not recommended (20). There is no data on the efficacy in primary antibody defects at present, but it is possible to hypothesize that, in response to the adjuvated vaccine, specific T cells might be able to undergo activation and terminal differentiation, thus envisioning a potential benefit of subunit vaccination.

## Insight on SARS-CoV-2

### The Adaptive Immune Responses

Effective vaccines against SARS-CoV-2 are being administered worldwide with the aim of terminating the COVID-19 pandemics. As for all immunizations, the efficacy has been linked to the production of specific antibodies, which increase in response to all vaccines in use (21). It should be underlined that in the study population of the pre-approval studies, no patients with primary immune deficiencies were included (11). In immunocompetent subjects, the level of neutralizing antibodies is highly predictive of immune protection, and mRNA vaccination generated robust, multi-component humoral and cellular immune memory to SARS-CoV-2 for at least 6 months after mRNA vaccination (22). Moreover, boosting of pre-existing immunity with mRNA vaccines in SARS-CoV-2 recovered individuals increased antibody responses (23). While immunization shots and subsequent boosters raised antibody levels in immunocompetent subjects, it is unclear which CVID patients might reach protection, have a reduced infectious risk and disease severity. Data on immunogenicity of SARS-CoV-2 vaccine in patients with CVID are still limited. Since vaccination became available, Italian CVID patients, as well as CVID from other countries, were immunized mostly with mRNA COVID-19 vaccines. Four groups have recently reported immune responses to vaccines in patients with inborn errors of immunity, including CVID patients (24–27). In the study by Hagin et al., 70% of the adult patients with predominantly antibody deficiency developed specific humoral and T-cell responses after 2 doses of SARS-CoV-2 mRNA vaccine. About one third of CVID patients did not produce Spike-specific IgG, mainly including patients with low B cell number and reduced switched memory B cells. Two thirds of the patients had produced specific antibodies, in particular those belonging to the group with normal frequency of switched memory B cells (Euroclass smB+). The authors concluded that the GC reaction had a crucial role on protective antibody generation (24). Similarly, in the paper from Romano et al, 4 CVID patients developed neutralizing antibodies against SARS-CoV-2. The only patient who failed to have a substantial rise in post vaccination titers had a marked decrease in the frequency of circulating B cells (25). In a recent paper, a high percentage of fully immunized CVID patients developed anti-Spike IgG, at significantly lower levels than in the healthy control group (26). Our data (27) in CVID patients immunized with BNT162b2 SARS-CoV-2 mRNA vaccine contrasted with the high frequency of response reported in the cohorts illustrated above. We show that only 20% of CVID patients developed both anti-Spike IgG and IgA antibodies, and one patient responded with IgG only. Moreover, the level of antibodies in the few patients who produced specific IgG and IgA was significantly lower compared to vaccinated healthy donors. CVID patients who did not mount a detectable antibody response after immunization had lower frequency of switched memory B cells and lower serum IgA and IgM levels. In detail, 10% of those with low frequencies of switched memory B cells (<2%) showed a detectable humoral response. However, similarly to what observed in immunocompetent individuals, in some CVID patients who were previously infected with SARS-CoV-2, the IgG response was boosted by the subsequent immunization, showing that SARS-CoV-2 infection might effectively prime the immune response (28). Administration of BNT162b2 SARS-CoV-2 mRNA vaccine does not induce the production of specific SIgA in the mucosa of the respiratory tract in healthy individuals (29). It is therefore unlikely that vaccination may induce mucosal protection in CVID patients lacking SIgA. In order to protect SARS-CoV-2 CVID infected patient, passively infused SARS-CoV-2 monoclonal antibodies treatment was effective and well-tolerated in patients with Primary Antibody Defects (30).

The huge variation among the response rates reported (2) confirms that CVID includes a complex mixture of patients with different levels of immune impairment. In addition, as hypogammaglobulinemia is the hallmark of CVID, we still ignore whether the response to COVID vaccines will represent a durable or transient change of the B-cell repertoire in this category of patients. Whereas we expect that after the initial decline, antibody levels will reach a plateau in control subjects reflecting the establishment of the long-lived memory plasma cell pool, this may never happen in CVID and antibodies may rapidly become undetectable. For this reason, although antibody measurement, month after vaccination, is neither indicated nor useful for the majority of vaccinees, the change in the concentration of Spike specific antibodies may represent an important measure of the ability of CVID patients to prevent SARS-CoV-2 severe disease.

Additional information can be obtained by combining the analysis of the B cell phenotype with the detection of Spike and RBD-specific B cells. We concentrated our study on the B cell response, because, independently of whether in each single patient CVID is caused by defects of B, T or innate cells, the final outcome is the lack of antibody responses measurable by immunoglobulin concentration and B cell phenotyping. High specificity and affinity are the most important characteristics of protective MBCs, generated by the adaptive immune system in response to infection or vaccination (31). While the natural course of COVID-19 is primarily characterized by the function of the innate immune system, with a secondary involvement of T and B cells, SARS-CoV2 vaccines are designed to force the adaptive immune system to generate neutralizing antibodies and Spike antigen-specific memory B and T cells. Immunocompetent subjects responded to SARS-CoV-2 immunization by generating classical MBCs with high and low binding capacity for Spike and activated MBCs (32). Moreover, they also generated few ATM B cells with low binding capacity and plasmablasts with low and high binding capacity. In CVID, impaired differentiation of mature post-GC B-cells, with severely reduction of switched MBCs and plasmablast/plasma cells are the most consistent defects. Impaired maturation of B-cells might occur also at the pre-GC stage, leading to a strongly reduced number of B cells in the periphery (33). Upon vaccination, CVID patients did not generate classical and activated MBCs, but SARS-CoV-2 vaccination induced only ATM B cells with low binding capacity to Spike protein (27). It has been suggested that ATM B cells are short-lived activated cells, in the process of differentiating into plasma cells. ATM B cells may be produced by extra-follicular reactions or failure of the GC reactions (34). High affinity plasmablasts were not produced in CVID, while one third of the patients generated low affinity plasmablasts. None of the CVID patients generated memory B cells specific for the receptor binding domain (RBD) of SARS-CoV-2, indicating the incapability of CVID B-cells to undergo somatic mutation and affinity maturation in the GC indispensable for the production of neutralizing antibodies. This impairment, associated to the generation of atypical memory or classical memory B cells with low affinity for the Spike protein is the basis to hypothesize a sub-optimal and transient humoral immune response after vaccination in CVID patients.

Remarkably, CVID patient convalescents from COVID-19 generated classical Spike-specific MBCs after vaccination (28). Since natural infection responses are boosted by subsequent immunization, the comparison of immune responses generated by the vaccine and the infection will be important to shed light on the difference between an antigen-driven response and an infection-driven response.

The role of T cells in antiviral responses and formation of immunological memory in general is well-recognized. SARS-CoV-2-specific T cells have been identified in all T cell subsets, TCM, TEM, and TEMRA subsets in immunocompetent individuals. Indeed, several studies have demonstrated T cell memory and effector responses against a broad selection of epitopes from SARS-CoV-2, as well as cross-reactive responses in unexposed individuals (35). The majority of the immunocompetent subjects developed T-cell responses following immunization, with variable results observed only in aged individuals (36).

Spike-specific T-cells responses were induced in CVID patients with a variable frequency (37, 38), differently from T cells produced after multiple exposures to influenza viral antigen following immunization or infection. In a recent study, the vast majority of CVID patients had S1-specific T cells compared after two doses of immunization with mRNA vaccine (39). In our study (27), differently from influenza immunization, poor Spike-specific T-cell responses were generated by immunization. SARS-CoV-2 is a pathogen never encountered before, since SARS-CoV-2 Spike and the RBD domains are distinct from the Spike proteins of most members of the coronavirus family (40). Then, it is possible that the first antigenic stimulation was not sufficient to induce an early T-cell response in CVID.

### The Innate Immunity Responses

Cells of the innate immune system play an essential role in early protection against infectious disease. To pathogens for which there is no preexisting immunity, the innate immune system is activated with the intent of limiting the infection while the adaptive immune response develops slowly and needs two weeks to generate the most specific and effective defensive tools: high affinity antibodies and memory B and T cells (41). After two years from the beginning of the pandemic, it remains unclear whether the condition of primary antibody deficiency is a predisposing or a protective factor for SARS-CoV-2 infection (42). Moreover, treatments for the immune deficiency status might also interfere with the disease progression and to the response to SARS-CoV-2 immunization. It should be remembered here that patients with antibody deficiencies receive monthly immunoglobulin replacement therapy to substitute the lack of antibody production. The pool of immunoglobulin might include cross-reacting antibodies to SARS-CoV-2, and might act by modulating monocytes and macrophages activities, even when administered at replacement dosages (43). We suggested (44) that antibody deficiency patients might be protected from severe COVID-19 by loss of Interleukin-6 and by impaired toll-like receptor (TLR) pathway activation. TLR pathway activation is impaired in CVID, particularly activation by TLR7 and TLR9, involved in antiviral innate immune responses and in the cytokine storm leading to an exaggerated activation of lung residing immune cells (45). Thus, CVID patients might be partially protected against the dangerous macrophage hyperactivation resulting in cytokine storm. On the contrary, CVID patients with an underlying inflammatory chronic lung disease have a worse COVID-19 prognosis (46). CVID-associated immune dysregulation is a Th1-mediated inflammatory process driven by the IFN-γ pathway (47) and by a persistent activation on innate immunity (48), possibly due to the activation of IFN-γ:STAT1:BAFF axis leading to a dysregulated B cell responses (49). The interferon signature in CVID has been also linked to the expansion of circulating IFN-γ-producing innate lymphoid cells (50). Differently from COVID-19, the highly augmented IFN signaling and cytotoxic signature has not been detected after vaccination with the SARS-CoV-2 mRNA vaccines (51). In particular, the upregulation of gene signature associated with type I and type II IFN production was not observed in the immunized subjects, suggesting an adaptive immunity maturation in the absence of IFN signaling.

## Conclusions

Despite the antibody deficiency, T-cell immunity is thought to be largely intact in many patients with CVID. For this reason, immunologists recommend routine administration of multiple vaccines with the exception of those containing attenuated viruses. Immunization of CVID patients against SARS-CoV-2 offered the possibility to analyze how defective mechanisms impact the immune response to a novel antigen. Discrepancy in the results published on antibody responses after SARS-CoV-2 immunization in CVID might be due to the heterogeneity of CVID populations enrolled or to different vaccination protocols. In CVID as well as in immunocompetent subjects, the nature of B- and T-cell responses differs dramatically between infected and vaccinated individuals, suggesting that inflammatory responses associated with infection influence the trajectory of the adaptive immune response. Moreover, the observation that the humoral immune response induced by natural infection was significantly enhanced by subsequent immunization underlines the need to immunize COVID-19 convalescent CVID patients after recovery. The search for a strategy to elicit an adequate protective immune response post-vaccination in CVID patients is necessary. This might include the changes of vaccination schedules, such as administration of multiple doses or to booster with heterologous vaccine preparation. In order to achieve protection of patients with immunodeficiency, new strategies may be necessary, such as the development of different types of vaccines, including those with inactivated viruses, high content of antigens, or with adjuvant. At present, we do not know whether CVID patients might require multiple doses or combinations of SARS-CoV-2 vaccines to obtain protection. Since reinfection with SARS-CoV-2 after vaccination or previous infection has been documented in CVID, at present SARS-CoV-2 positive CVID patients might benefit from the new preventing strategy based on administration of anti-SARS-CoV-2 monoclonal antibodies, and - in the next future - by the newly produced lots of gamma globulins for substitution therapy that will contain Spike-specific IgG.

The results obtained by the administration of mRNA vaccines against a virus never encountered by humans before has given us the possibility to study the different immune responses of the complex community of CVID patients and compare them to healthy controls. Thanks to the availability of new tools and methods, developed because of the COVID-19 pandemic, we are able not only to measure antibodies but also antigen-specific B and T cells. We should now combine the results obtained with the complete vaccine cycle and the booster dose, completed by a follow-up to measure the persistence of specific antibody and memory B cells. On the basis of this study it will be possible to distinguish the group of CVID patients who may benefit from vaccination and also pin-point the specific step of the immune response defective in each individual.

## Data Availability Statement

The original contributions presented in the study are included in the article/supplementary material. Further inquiries can be directed to the corresponding author.

## Author Contributions

IQ and RC elaborated data and wrote the manuscript. FL revised and commented the manuscript. All authors defined the conclusion. All authors contributed to the article and approved the submitted version.

## Funding

The work was funded by GSK project: Call for Prevention, Italian Ministry of Health COVID-2020-12371817 grant and grant “5 per mille, 2021” to RS. The funder was not involved in the study design, collection, analysis, interpretation of data, the writing of this article or the decision to submit it for publication.

## Conflict of Interest

The authors declare that the research was conducted in the absence of any commercial or financial relationships that could be construed as a potential conflict of interest.

## Publisher’s Note

All claims expressed in this article are solely those of the authors and do not necessarily represent those of their affiliated organizations, or those of the publisher, the editors and the reviewers. Any product that may be evaluated in this article, or claim that may be made by its manufacturer, is not guaranteed or endorsed by the publisher.

## References

[1] Tangye SG Al-Herz W Bousfiha A Bousfiha A Chatila T Cunningham-Rundles C. Human Inborn Errors of Immunity: 2019 Update on the Classification From the International Union of Immunological Societies Expert Committee. J Clin Immunol (2020) 40:24–64. doi: 10.1007/s10875-019-00737-x 31953710 PMC7082301

[2] Bonilla FA Barlan I Chapel H. Costa-Carvalho BT Cunningham-Rundles C de la Morena MT. International Consensus Document (ICON): Common Variable Immunodeficiency Disorders. J Allergy Clin Immunol Pract (2016) 4:38–59. doi: 10.1016/j.jaip.2015.07.025 26563668 PMC4869529

[3] Ponsford MJ Price C Farewell D Greene G Moore C Perry M. Increased Respiratory Viral Detection and Symptom Burden Among Patients With Primary Antibody Deficiency: Results From the BIPAD Study. J Allergy Clin Immunol Pract (2020) 9:735–44.e6. doi: 10.1016/j.jaip.2020.08.016 PMC7442926 32841749

[4] Pulvirenti F Cinetto F Milito C Bonanni L Pesce AM Leodori G. Health-Related Quality of Life in Common Variable Immunodeficiency Italian Patients Switched to Remote Assistance During the COVID-19 Pandemic. J Allergy Clin Immunol Pract (2020) 8:1894–9.e2. doi: 10.1016/j.jaip.2020.04.003 PMC7195351 32278865

[5] Milito C Lougaris V Giardino G Punziano A Vultaggio A Carrabba M. Clinical Outcome, Incidence, and SARS-CoV-2 Infection-Fatality Rates in Italian Patients With Inborn Errors of Immunity. J Allergy Clin Immunol Pract (2021) 9:2904–6.e2. doi: 10.1016/j.jaip.2021.04.017 PMC8059325 33894392

[6] Meyts I Bucciol G Quinti I Neven Bénédicte Fischer A Seoane E. Coronavirus Disease 2019 in Patients With Inborn Errors of Immunity: An International Study. J Allergy Clin Immunol (2021) 147:520–31. doi: 10.1016/j.jaci.2020.09.010 PMC7832563 32980424

[7] Gram Grammatikos A Donati M Johnston SL Gompels MM. Peripheral B Cell Deficiency and Predisposition to Viral Infections: The Paradigm of Immune Deficiencies. Front Immunol (2021) 12:731643. doi: 10.3389/fimmu.2021.731643 34527001 PMC8435594

[8] Khoenkhoen S Ádori M Pedersen GK Karlsson Hedestam GB. Flow Cytometry-Based Protocols for the Analysis of Human Plasma Cell Differentiation. Front Immunol (2020) 11:571321. doi: 10.3389/fimmu.2020.571321 33133085 PMC7550473

[9] Russo R Andolfo I Lasorsa VA Cantalupo S Marra R Frisso G. The TNFRSF13C H159Y Variant Is Associated With Severe COVID-19: A Retrospective Study of 500 Patients From Southern Italy. Genes (Basel) (2021) 12:881. doi: 10.3390/genes1206088 34201032 PMC8226789

[10] Schmidt A Peters S Knaus A Sabir H Hamsen F Maj C. TBK1 and TNFRSF13B Mutations and an Autoinflammatory Disease in a Child With Lethal COVID-19. NPJ Genom Med (2021) 6:55. doi: 10.1038/s41525-021-00220-w 34210994 PMC8249618

[11] D’Amelio R Asero R Cassatella MA Laganà B Lunardi C Migliorini P. Anti-COVID-19 Vaccination in Patients With Autoimmune-Autoinflammatory Disorders and Primary/Secondary Immunodeficiencies: The Position of the Task Force on Behalf of the Italian Immunological Societies. Biomed (2021) 9:1163. doi: 10.3390/biomedicines9091163 PMC8465958 34572349

[12] Romberg N Le Coz C Glauzy S Schickel JN Trofa M Nolan BE. Patients With Common Variable Immunodeficiency With Autoimmune Cytopenias Exhibit Hyperplastic Yet Inefficient Germinal Center Responses. J Allergy Clin Immunol (2019) 143:258–65. doi: 10.1016/j.jaci.2018.06.012 PMC6400323 29935219

[13] Kaneko N Kuo HH Boucau J Farmer JR Allard-Chamard H Mahajan VS. Loss of Bcl-6-Expressing T Follicular Helper Cells and Germinal Centers in COVID-19. Cell (2020) 183(1):143–57.e13. doi: 10.1016/j.cell.2020.08.025 PMC7437499 32877699

[14] Woodruff MC Ramonell RP Nguyen DC Cashman KS Saini AS Haddad NS. Extrafollicular B Cell Responses Correlate With Neutralizing Antibodies and Morbidity in COVID-19. Nat Immunol (2020) 21(12):1506–16. doi: 10.1038/s41590-020-00814-z PMC7739702 33028979

[15] Milito C Soccodato V Collalti G Lanciarotta A Bertozzi I Rattazzi M. Vaccination in PADs. Vaccines (Basel) (2021) 9:626. doi: 10.3390/vaccines9060626 34207916 PMC8230118

[16] Pulvirenti F Milito C Cavaliere FM Mezzaroma I Cinetto F Quinti I. IGA Antibody Induced by Immunization With Pneumococcal Polysaccharides Is a Prognostic Tool in Common Variable Immune Deficiencies. Front Immunol (2020) 11:1283. doi: 10.3389/fimmu.2020.01283 32695106 PMC7336165

[17] Guevara-Hoyer K Gil C Parker AR Williams LJ Orte C Rodriguez de la Peña A. Measurement of Typhim Vi IgG as a Diagnostic Tool to Determine Anti-Polysaccharide Antibody Production Deficiency in Children. Front Immunol (2019) 10:654. doi: 10.3389/fimmu.2019.00654 31001267 PMC6455213

[18] Kostinova AM Akhmatova NK Latysheva EA Dagil YA Klimova SV. Assessment of Immunogenicity of Adjuvanted Quadrivalent Inactivated Influenza Vaccine in Healthy People and Patients With Common Variable Immune Deficiency. Front Immunol (2020) 11:1876. doi: 10.3389/fimmu.2020.01876 32973775 PMC7466564

[19] Friedmann D Goldacker S Peter HH Warnatz K. Preserved Cellular Immunity Upon Influenza Vaccination in Most Patients With Common Variable Immunodeficiency. J Allergy Clin Immunol Pract (2020) 8:2332–2340.e5. doi: 10.1016/j.jaip.2020.04.019 32330665

[20] Harbecke R Cohen JI Oxman MN. Herpes Zoster Vaccines. J Infect Dis (2021) 224:S429–42. doi: 10.1093/infdis/jiab387 PMC8482024 34590136

[21] Sharif N Alzahrani KJ Ahmed SN Dey SK. Efficacy, Immunogenicity and Safety of COVID-19 Vaccines: A Systematic Review and Meta-Analysis. Front Immunol (2021) 12:714170. doi: 10.3389/fimmu.2021.714170 34707602 PMC8542872

[22] Goel RR Painter MM Apostolidis SA Mathew D Meng W Rosenfeld AM. mRNA Vaccines Induce Durable Immune Memory to SARS-CoV-2 and Variants of Concern. Science (2021) 374(6572):eabm0829. doi: 10.1126/science.abm0829 PMC9284784 34648302

[23] Naaber P Tserel L Kangro K Epp Seppb E Jurjenson V Adamson A. Dynamics of Antibody Response to BNT162b2 Vaccine After Six Months: A Longitudinal Prospective Study. Lancet Reg Health Eur (2021) 10:100208. doi: 10.1016/j.lanepe.2021.100208 34514454 PMC8418937

[24] Hagin D Freund T Navon M Halperin T Adir D Marom R. Immunogenicity of Pfizer-BioNTech COVID-19 Vaccine in Patients With Inborn Errors of Immunity. J Allergy Clin Immunol (2021) 148:739–49. doi: 10.1016/j.jaci.2021.05.029 PMC8168345 34087242

[25] Romano C Esposito S Donnarumma G Marrone A. Detection of Neutralizing Anti-Severe Acute Respiratory Syndrome Coronavirus 2 Antibodies in Patients With Common Variable Immunodeficiency After Immunization With Messenger RNA Vaccines. Ann Allergy Asthma Immunol (2021) 127:499–501. doi: 10.1016/j.anai.2021.07.026 34352358 PMC8327608

[26] Delmonte OM Bergerson JRE Burbelo PD Durkee-Shock JR Dobbs K Bosticardo M. Antibody Responses to the SARS-CoV-2 Vaccine in Individuals With Various Inborn Errors of Immunity. J Allergy Clin Immunol (2021) 148:1192–7. doi: 10.1016/j.jaci.2021.08.016 PMC8418380 34492260

[27] Fernandez Salinas A Piano Mortari E Terreri S Quintarelli C Pulvirenti F Di Cecca S. SARS-CoV-2 Vaccine Induced Atypical Immune Responses in Antibody Defects: Everybody Does Their Best. J Clin Immunol (2021) 41:1709–22. doi: 10.1007/s10875-021-01133-0 PMC8527979 34669144

[28] Pulvirenti F Fernandez Salinas A Milito C Terreri S Piano Mortari E Quintarelli C. B Cell Response Induced by SARS-CoV-2 Infection Is Boosted by the BNT162b2 Vaccine in Primary Antibody Deficiencies. Cells (2021) 2915:2915–30. doi: 10.3390/cells10112915 PMC8616496 34831138

[29] Quinti I Piano Mortari E Fernandez Salinas A Milito C Carsetti R. IgA Antibodies and IgA Deficiency in SARS-CoV-2 Infection. Front Cell Infect Microbiol (2021) 11:655896. doi: 10.3389/fcimb.2021.655896 33889552 PMC8057809

[30] Pulvirenti F Milito C Cinetto F Salinas AF Terreri S Mortari EP. SARS-CoV-2 Monoclonal Antibody Combination Therapy in Patients With COVID-19 and Primary Antibody Deficiency. J Infect Dis (2021) 554:1–5. doi: 10.1093/infdis/jiab554 PMC8689914 34746954

[31] Tangye SG Tarlinton DM. Memory B Cells: Effectors of Long-Lived Immune Responses. Eur J Immunol (2009) 39:2065–75. doi: 10.1002/eji.200939531 19637202

[32] Piano Mortari E Russo C Vinci MR Terreri S Fernandez Salinas A Piccioni L. Highly Specific Memory B Cells Generation After the 2nd Dose of BNT162b2 Vaccine Compensate for the Decline of Serum Antibodies and Absence of Mucosal IgA. Cells (2021) 10:2541. doi: 10.3390/cells10102541 34685521 PMC8533837

[33] Del Pino-Molina L López-Granados E Lecrevisse Q Torres Canizales J Pérez-Andrés M Blanco E. Dissection of the Pre-Germinal Center B-Cell Maturation Pathway in Common Variable Immunodeficiency Based on Standardized Flow Cytometric EuroFlow Tools. Front Immunol (2021) 11:603972. doi: 10.3389/fimmu.2020.603972 33679693 PMC7925888

[34] Braddom AE Batugedara G Bol S Bunnik EM. Potential Functions of Atypical Memory B Cells in Plasmodium-Exposed Individuals. Int J Parasitol (2020) 50:1033–42. doi: 10.1016/j.ijpara.2020.08.003 PMC7666103 32987039

[35] Niessl J Sekine T Buggert M. T Cell Immunity to SARS-CoV-2. Semin Immunol (2021) 55:101505. doi: 10.1016/j.smim.2021.101505 34711489 PMC8529278

[36] Pegu A O’Connell SE Schmidt SD O’Dell S Talana CA Lai L. Durability of mRNA-1273 Vaccine-Induced Antibodies Against SARS-CoV-2 Variants. Sci (2021) 373:1372–7. doi: 10.1126/science.abj4176 PMC8691522 34385356

[37] Ameratunga R Longhurst H Steele R Lehnert K Leung E Brooks AES. Common Variable Immunodeficiency Disorders, T-Cell Responses to SARS-CoV-2 Vaccines, and the Risk of Chronic COVID-19. J Allergy Clin Immunol Pract (2021) 9:3575–83. doi: 10.1016/j.jaip.2021.06.019 PMC8230758 34182162

[38] Gupta S Su H Narsai T Agrawal S. SARS-CoV-2-Associated T-Cell Responses in the Presence of Humoral Immunodeficiency. Int Arch Allergy Immunol (2021) 182:195–209. doi: 10.1159/000514193 33486489 PMC7900460

[39] Arroyo-Sánchez D Cabrera-Marante O Laguna-Goya R Almendro-Vázquez P Carretero O Gil-Etayo FJ. Immunogenicity of Anti-SARS-CoV-2 Vaccines in Common Variable Immunodeficiency. J Clin Immunol (2021), 1–13. doi: 10.1007/s10875-021-01174-5 PMC8596355 34787773

[40] Steiner S Sotzny F Bauer S Na IK Schmueck-Henneresse M Corman VM. HCoV- and SARS-CoV-2 Cross-Reactive T Cells in CVID Patients. Front Immunol (2020) 11:607918. doi: 10.3389/fimmu.2020.607918 33424856 PMC7785785

[41] Carsetti R Zaffina S Piano Mortari E Terreri S Corrente F Capponi C. Different Innate and Adaptive Immune Responses to SARS-CoV-2 Infection of Asymptomatic, Mild, and Severe Cases. Front Immunol (2020) 11:610300. doi: 10.3389/fimmu.2020.610300 33391280 PMC7772470

[42] Babaha F Rezaei N. Primary Immunodeficiency Diseases in COVID-19 Pandemic: A Predisposing or Protective Factor? Am J Med Sci (2020) 360:740–1. doi: 10.1016/j.amjms.2020.07.027 PMC7388814 32773108

[43] Quinti I Mitrevski M. Modulatory Effects of Antibody Replacement Therapy to Innate and Adaptive Immune Cells. Front Immunol (2017) 8:697. doi: 10.3389/fimmu.2017.00697 28670314 PMC5472665

[44] Quinti I Lougaris V Milito C Cinetto F Pecoraro A Mezzaroma I. A Possible Role for B Cells in COVID-19? Lesson From Patients With Agammaglobulinemia. J Allergy Clin Immunol (2020) 146:211–3.e4. doi: 10.1016/j.jaci.2020.04.013 PMC7175894 32333914

[45] Yu JE Knight AK Radigan L Marron TU Zhang L Sanchez-Ramon S. Toll-Like Receptor 7 and 9 Defects in Common Variable Immunodeficiency. J Allergy Clin Immunol (2009) 124:349–56.e3563. doi: 10.1016/j.jaci.2009.05.019 PMC2908501 19592080

[46] Milito C Soccodato V Auria S Pulvirenti F Quinti I. COVID-19 in Complex Common Variable Immunodeficiency Patients Affected by Lung Diseases. Curr Opin Allergy Clin Immunol (2021) 21:535–44. doi: 10.1097/ACI.0000000000000789 34580250

[47] Hultberg J Ernerudh J Larsson M Nilsdotter-Augustinsson Å Nyström S. Plasma Protein Profiling Reflects T_H_1-Driven Immune Dysregulation in Common Variable Immunodeficiency. J Allergy Clin Immunol (2020) 146:417–28. doi: 10.1016/j.jaci.2020.01.046 32057767

[48] Tsinti G Makris D Germenis AE Speletas M. Persistent Activation of Innate Immunity in Patients With Primary Antibody Deficiencies. J Immunol Res (2020) 2020:8317671. doi: 10.1155/2020/8317671 33274244 PMC7695510

[49] Matson EM Abyazi ML Bell KA Hayes KM Maglione PJ. B Cell Dysregulation in Common Variable Immunodeficiency Interstitial Lung Disease. Front Immunol (2021) 11:622114. doi: 10.3389/fimmu.2020.622114 33613556 PMC7892472

[50] Maglione PJ Cols M Cunningham-Rundles C. Dysregulation of Innate Lymphoid Cells in Common Variable Immunodeficiency. Curr Allergy Asthma Rep (2017) 17:77. doi: 10.1007/s11882-017-0746-6 28983810 PMC5897894

[51] Ivanova EN Devlin JC Buus TB Koide A Shwetar J Cornelius A. Discrete Immune Response Signature to SARS-CoV-2 mRNA Vaccination Versus Infection. medRxiv (2021). doi: 10.1101/2021.04.20.21255677

